# Daphnoretin inhibits glioblastoma cell proliferation and metastasis via PI3K/AKT signaling pathway inactivation

**DOI:** 10.7150/jca.98915

**Published:** 2024-09-09

**Authors:** Jiaming Lei, Hong Zhou, Shijiao Cheng, Wenwen Yu, Meiting Yang, Li Lin

**Affiliations:** 1 Key Laboratory of Environmental Related Diseases and One Health, School of Basic Medical Sciences, Xianning Medical College, Hubei University of Science and Technology, Xianning, 437100, China.; 2 Department of Medicine, Hubei University of Science and Technology, Xianning, 437100, China.

**Keywords:** daphnoretin, glioblastoma, PI3K/AKT, apoptosis, proliferation

## Abstract

Glioblastoma (GBM) was the most malignant intracranial tumor with high mortality rates and invariably poor prognosis due to its limited clinical treatments. The urgent need to develop new therapeutic drugs for GBM treatment is evident. As a coumarin derivative, daphnoretin's favorable pharmacological activities have been widely documented. However, the potential inhibitory effects of daphnoretin on GBM have not been explored. In this study, we aimed to investigate the effects of daphnoretin on GBM and elucidate its anti-GBM mechanisms for the first time. It was observed that daphnoretin inhibited GBM cell proliferation, migration, and invasion in vitro and suppressed tumor growth without significant drug toxicity in GBM xenograft tumor models *in vivo*. Mechanistically, daphnoretin was predicted to target the PI3K/AKT signaling pathway through network pharmacology and molecular docking analysis. Subsequently, it was further verified by Biacore assay for surface plasmon resonance (SPR) experiments. Experimentally, daphnoretin induced apoptosis in GBM cells via the PI3K/AKT signaling pathway. Moreover, the effects of daphnoretin on GBM cells could be reversed by the AKT activator SC79. These results suggest that daphnoretin holds potential as a therapeutic drug against GBM and provides new insights into GBM treatment.

## 1. Introduction

Glioblastoma (GBM) is the most common and malignant primary brain tumor in adults. Despite aggressive management involving maximal safe surgical resection followed by external beam radiation therapy with concomitant and adjuvant temozolomide (TMZ), approximately 90% of WHO grade IV gliomas (GBM) recur locally within 2 years 1, 2. Currently, no standard of care exists for patients with recurrent GBM, despite available post-neurosurgical treatment options such as temozolomide, radiotherapy, and some targeted drugs. Consequently, the prognosis for GBM patients remains bleak 3. Thus, exploring potential drugs for clinical treatment is urgently needed.

In recent years, with the rapid development of natural medicine, traditional Chinese medicine monomers have garnered increasing attention due to their potent antitumor effects 4. Daphnoretin, a primary active ingredient of Daphne giraldii Nitsche, exhibits various pharmacological effects, including antitumor, antioxidative, and anti-inflammatory properties 5. Daphnetin, chemically known as 7-hydroxyl-6-methoxy-3,7'-dicoumarylether, with molecular formula of C_19_H_12_O_7_ and molecular weight of 352.29. Daphnetin has shown a broad application prospect in the field of medicine due to its unique biochemical and molecular properties 6. Daphnoretin has attracted significant interest due to these unique pharmacological effects. Studies have demonstrated its substantial antitumor activity across various cancers, including inhibition of proliferation, induction of apoptosis, and cell cycle arrest 7-9. However, research on the therapeutic effects and mechanisms of daphnoretin in GBM remains limited. As a coumarin derivative, daphnoretin possesses diverse pharmacological activities, yet its potential inhibitory effect on GBM has not been thoroughly investigated. Our findings suggest that daphnoretin may offer a promising therapeutic option for GBM patients.

In the present study, we extensively investigated daphnoretin's effects on GBM. Daphnoretin exhibits pharmacological activities that inhibit GBM cell proliferation, colony formation, migration, and invasion *in vitro*, and suppresses tumor growth with no significant adverse effects *in vivo*.

In summary, this study systematically evaluates the potential of daphnoretin in GBM treatment and explores its anti-tumor mechanism. We aim to identify new drug candidates and treatment strategies for GBM, ultimately improving therapeutic outcomes and enhancing patients' quality of life.

## 2. Materials and Methods

### 2.1 Materials and reagents

Daphnoretin (CAS: 2034-67-9) and Temozolomide (TMZ) (CAS: 85622-93-1) were purchased from Chinese Shanghai Yuanye Bio-Technology Co., Ltd and Chinese Shanghai Aladdin Biochemical Technology Co., Ltd, respectively. AKT agonist SC79 (CAS: 305834-79-1) was obtained from MedChemExpress Co., Ltd. All chemicals were diluted with dimethyl sulfoxide (DMSO) (CAS: 67-68-5; Sigma-Aldrich Corp., Darmstadt, Germany), unless otherwise specified. Human GBM cell lines U87 and U251, and human brain glial cell line (normal) HEB were procured from Procell Life Science & Technology Co., Ltd (Wuhan, Hubei, China). U87, U251, and HEB cells were cultured in high-glucose Dulbecco's modified Eagle's medium (DMEM) (Gibco, Waltham, MA, USA), supplemented with 10% fetal bovine serum (FBS) (Gibco, Waltham, MA, USA) and 1% penicillin-streptomycin (Gibco, Waltham, MA, USA). All cells were maintained in a 5% CO2 humidified incubator at 37°C under 95% air.

### 2.2 Cell viability assay

Cell viability was assessed using the MTT assay to determine the half maximal inhibitory concentration (IC50) values. U87, U251, and HEB cells (9 × 10^4^) were seeded into 96-well plates (Costar, Corning, NY, USA) and cultured for 24 and 48 hours in the incubator. Cells were treated with inhibitors or various concentrations of daphnoretin. Subsequently, 10 μL of MTT solution was added to each well, followed by a 4-hour incubation period. After incubation, 100 μL of DMSO was added to dissolve the formazan crystals. The plates were vortexed for 10 minutes at room temperature, and absorbance was measured at 450 nm using the Bio-Tek ELx800 Multi-Mode Reader (BioTek Instruments, Inc., Winooski, VT, USA).

### 2.3 Colony formation assay

U87 and U251 cells were seeded at 1,000 cells per well in 6-well culture plates (Costar, Corning, NY, USA) and treated with DMSO or daphnoretin (10 and 20 μM) for 7-14 days. Cells were fixed with 4% paraformaldehyde for 15 minutes and stained with 0.5% crystal violet solution for another 15 minutes. Colonies (>100 cells/colony) were manually counted.

### 2.4 5‑Ethynyl‑2'‑deoxyuridine (EdU) incorporation assay

GBM cell proliferation was assessed using the Cell-Light EdU kit (C0071S, Beyotime, Shanghai, China). U87 and U251 cells (5 × 10^3^) were seeded in 24-well plates (Costar, Corning, NY, USA) for the EdU assay. Cells were treated with daphnoretin at concentrations of 0, 10, 20, and 40 μM. EdU reagent (10 μM) was added to cells during DNA synthesis for 2 hours. After fixation with 4% formaldehyde and permeabilization with 0.5% Triton X-100, cell nuclei were stained with Hoechst 33342. Images were captured using an X71 fluorescence microscope (200× magnification; Olympus Corporation, Tokyo, Japan) in five randomly selected fields.

### 2.5 Wound healing assay

U87 and U251 cells in the logarithmic growth phase were detached, resuspended, and 3 × 10^5^ cells were seeded per well in 6-well plates (Costar, Corning, NY, USA). When cells reached 95% confluence, a 200 μL pipette tip was used to create scratches. After washing twice with PBS, cells were treated with daphnoretin solutions at different concentrations. Images were taken at various time points. After 24 hours, cells were fixed with 4% paraformaldehyde, washed with PBS, and photographed.

### 2.6 Cell migration and invasion assays

Cells (5 × 10^4^) in 200 μL serum-free medium were seeded into the upper chamber of a 24-well Transwell insert (Costar, Corning, NY, USA). The lower chamber was filled with 600 μL of DMEM supplemented with 10% FBS and the indicated concentrations of daphnoretin. For invasion assays, Matrigel (1:8 dilution; Corning BioCoat, #356234, NY, USA) was applied to the bottom of the Transwell insert before cell seeding. After 24 hours of incubation, cells that migrated or invaded through the membrane were fixed with 4% paraformaldehyde (Solarbio, Beijing, China) for 20 minutes and stained with 1% crystal violet for another 20 minutes. Stained cells were counted under an X71 fluorescence microscope (Olympus Corporation, Tokyo, Japan).

### 2.7 Reactive oxygen species (ROS) analysis

Intracellular ROS levels were assessed using 2',7′-dichlorofluorescin diacetate (DCFDA; Sigma-Aldrich) to measure its fluorescent oxidized product, dichlorofluorescein (DCF). U251 and U87 cells (2.5 × 10^4^) were seeded in 24-well plates (Costar, Corning, NY, USA) and pretreated with 3 μM N-acetyl-L-cysteine (Sigma-Aldrich) or vehicle for 6 hours. Following cotreatment with daphnoretin for 18 hours, cells were incubated with 2 μM DCFDA in fresh complete medium for 60 minutes at 37°C in the dark. DCF fluorescence intensity was measured using an Olympus Fluorescence Microscope. Additionally, cells were stained with the JC-1 probe (Biosharp, Shanghai, China) for 30 minutes at 37°C in the dark, washed with pre-cold PBS, and suspended in 500 µL PBS. The percentage of apoptotic cells was evaluated using the X71 fluorescence microscope (Olympus Corporation, Tokyo, Japan).

### 2.9 Annexin V-FITC/PI staining assay

U251 and U87 cells (2.5 × 10^4^) were treated with various concentrations of daphnoretin for 24 hours in 24-well plates (Costar, Corning, NY, USA). Cells were harvested, centrifuged, and resuspended in PBS. After re-suspending in binding buffer, cells were stained using the Annexin V-FITC/PI kit (C1067M, Beyotime, Shanghai, China) for 20 minutes at 37°C. The percentage of apoptotic cells was determined using an X71 fluorescence microscope (Olympus Corporation, Tokyo, Japan).

### 2.10 Western blot analysis

Following daphnoretin treatment, cells were collected and washed three times with ice-cold PBS. Cell lysis was performed using RIPA buffer (Beyotime Biotechnology Co., Ltd., Shanghai, China) supplemented with protease and phosphatase inhibitors for 30 minutes. After centrifugation, supernatants containing total cellular proteins were collected. Protein concentration was determined using a BCA assay kit. Samples were denatured with loading buffer at 100°C for 10 minutes and separated by 8%-12% SDS-PAGE. Proteins were then transferred onto PVDF membranes, which were subsequently blocked. After blocking, membranes were incubated overnight at 4°C with specific primary antibodies. Following primary antibody incubation, membranes were probed with HRP-conjugated secondary antibodies, and protein expression was visualized using ECL plus reagents (Beyotime Biotechnology Co., Ltd.). GAPDH was used as a loading control. Antibodies used were: Bcl-2 (1:1000, R23309), Bax (1:1000, R22708), Caspase 3 (1:1000, R23315), Cleaved-caspase 3 (1:1000, 341034), p-AKT (1:1000, 381555), AKT (1:1000, 342529), p-PI3K (1:1000, 301245), PI3K (1:1000, 343830), Caspase 9 (1:1000, 381336), Cleaved-caspase 9 (1:1000, 381238), and GAPDH (1:1000, 380626) (Zhengneng Biotechnology Co., Ltd., Chengdu, China).

### 2.11 GBM subcutaneous xenograft model

All animal experiments conducted in this study were approved by the Animal Ethics Committee of Hubei University of Science and Technology and were performed in accordance with institutional and international guidelines for animal care and use. GL261 cells were used to establish the GBM mouse xenograft model. Five-week-old male C57BL/6 mice weighing approximately [insert weight range] were subcutaneously injected with GL261 cells (5 × 10^6^ cells in 100 μL sterile PBS) on the back of each mouse. Once tumors reached an average volume of approximately 100 mm^3, mice were randomly assigned to the following groups: Control, TMZ group, low-dose daphnoretin group (Daphnoretin-L), and high-dose daphnoretin group (Daphnoretin-H). Tumor volumes were measured every 2 days using the formula: volume = a × b^2^ × 0.52 (where a is length and b is width in mm), and body weights were recorded daily. After 14 days, mice were euthanized, and xenograft samples from all groups were collected for further analysis.

### 2.12 Hematoxylin and eosin (HE) and immunohistochemistry (IHC) staining

Dehydrated tissues fixed in 4% paraformaldehyde were paraffin-embedded and sectioned into 4 μm slices. Tissue blocks were deparaffinized, rehydrated, and stained with HE (BL700A, Biosharp, Shanghai, China). The stained sections were mounted on coverslips and viewed under a microscope. For immunohistochemical staining, tissue sections were deparaffinized, rehydrated, and treated with 3% hydrogen peroxide to quench endogenous peroxidase activity. Antigen retrieval was performed in citrate buffer (pH 6.0). Sections were then blocked and permeabilized with 0.1% Triton X-100 and incubated overnight at 4°C with primary antibodies against CD31 (1:100, #3528, Cell Signaling Technology, MA, USA), Ki-67 (1:100, AF1738, Beyotime, Shanghai, China), PCNA (1:100, #13110, Cell Signaling Technology, MA, USA), p-AKT (1:100, 381555), AKT (1:100, 342529), p-PI3K (1:100, 301245), and PI3K (1:100, 343830) (Zhengneng Biotechnology Co., Ltd., Chengdu, China). After washing, sections were incubated with an HRP-conjugated secondary antibody for 1 hour at room temperature. Antibody binding was visualized using diaminobenzidine (DAB P0202, Beyotime, Shanghai, China) substrate, and sections were counterstained with hematoxylin (BL700A, Biosharp, Shanghai, China).

### 2.13 Network pharmacology analysis

The molecular structure of daphnoretin was obtained from PubChem (https://pubchem.ncbi.nlm.nih.gov/) in Simplified Molecular-Input Line-Entry System (SMILES) format. The SwissTargetPrediction database (www.swisstargetprediction.ch/) was utilized to predict potential targets of daphnoretin for Homo sapiens based on its chemical structure. Disease-related targets associated with glioblastoma (GBM) were retrieved from the GeneCards database (https://www.GeneCards.org/) using the keyword "glioblastoma". Predicted protein targets of daphnoretin against GBM were filtered and analyzed using Venny 2.1.0 (https://bioinfogp.cnb.csic.es/tools/venny/). Functional and pathway enrichment analyses of the overlapping daphnoretin-GBM targets were performed using R (v.3.6.3) software, incorporating Gene Ontology (GO) and Kyoto Encyclopedia of Genes and Genomes (KEGG) annotations. A protein-protein interaction (PPI) network of the identified targets was constructed using STRING (https://string-db.org/) and visualized with Cytoscape v3.8.0 software.

### 2.14 Molecular docking analysis

The compound name, molecular weight, and 3D structure of daphnoretin were obtained from the PubChem database, and the 3D structure was further downloaded from the RCSB Protein Data Bank (http://www.rcsb.org/). AutoDock software was used to prepare the ligands and proteins required for molecular docking. For each target protein, the crystal structure was processed to remove water molecules, hydrogenate, optimize amino acids, adjust energy parameters, and generate low-energy conformations of the ligand structure. Docking simulations were performed between daphnoretin and the selected target proteins, and the Binding Energy (kcal/mol) values were calculated to assess binding affinities. Lower binding energies indicate more stable interactions between the ligand and receptor. The results of molecular docking were analyzed using Discovery Studio software to visualize and interpret the binding modes, and the best docking poses based on binding energies were selected for further analysis.

### 2.15 Biacore assay for surface plasmon resonance (SPR) analysis

To validate the direct interaction between daphnoretin and AKT1, the Biacore T200 instrument (GE Healthcare) was utilized. Following expression, soluble protein was obtained through sonication and centrifugation, and subsequently incubated with anti-His beads to isolate the fusion protein. The protein was further concentrated using an ultrafiltration centrifuge tube, and its concentration was determined. For SPR analysis, a CM5 sensor chip was utilized. His-tagged AKT1 was immobilized via His antibody in parallel-flow channels of the CM5 sensor chip. Daphnoretin was injected at various concentrations into the flow system to assess its interaction with AKT1. Experiments were conducted in PBS buffer, and a dissociation time of 600 seconds was allowed. To correct for solvent effects, PBS with 5% DMSO was used.

### 2.16 Statistical analysis

Data are presented as mean ± SD. Statistical significance between control and experimental groups was assessed using paired Student's t-tests. A p-value < 0.05 was considered statistically significant.

## 3. Results

### 3.1 Daphnoretin inhibited GBM cells proliferation

**Figure 1:**
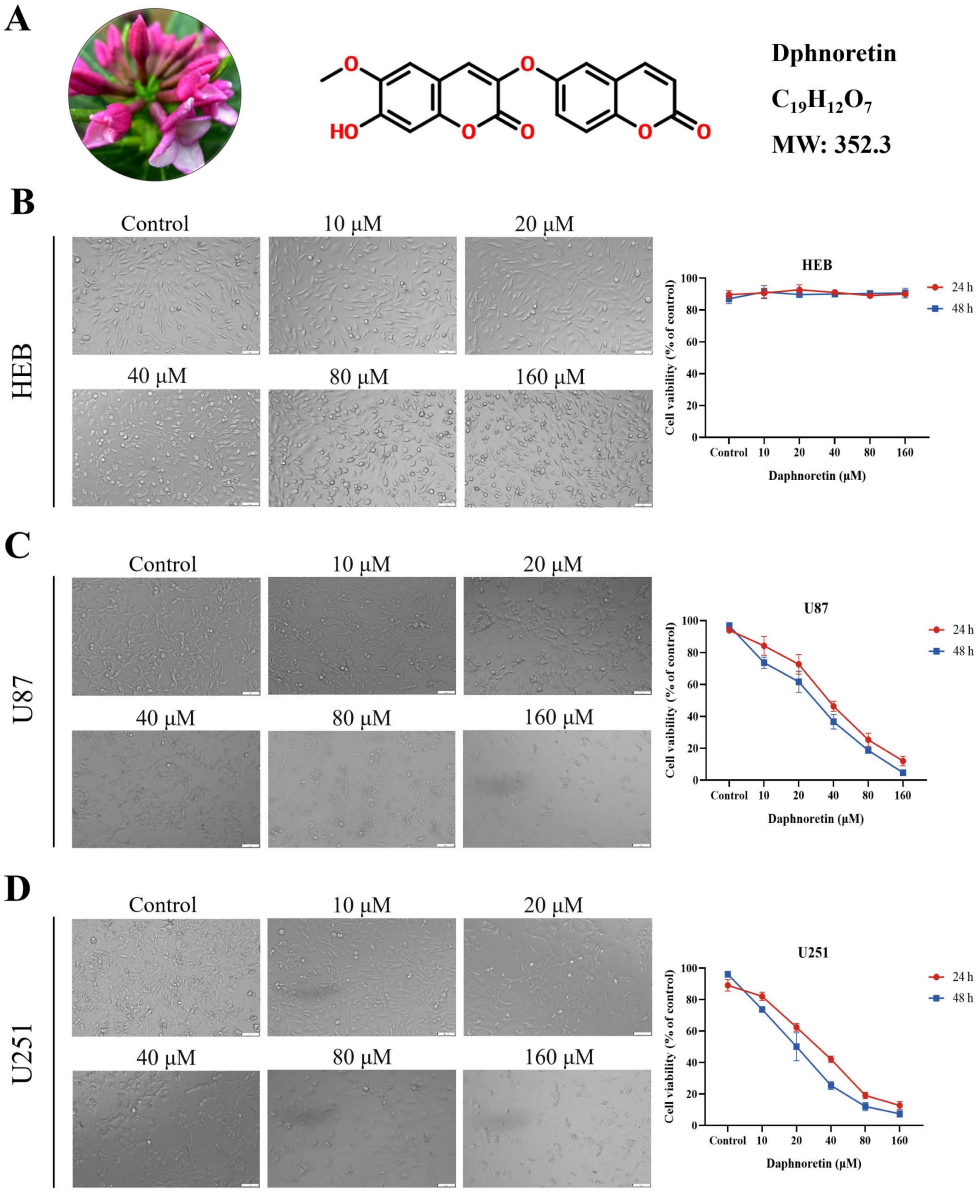
** Daphnoretin inhibits the viability of GBM cells.** (A) Medical material morphology, chemical structure, molecular formula and weight of daphnoretin. (B) Daphnoretin at different concentrations (0, 10, 20, 40, 80, 160 µM) treated HEB cells for 24 and 48 h. (C) Daphnoretin at different concentrations (0, 10, 20, 40, 80, 160 µM) treated HEB cells for 24 and 48 h. (D) Daphnoretin at different concentrations (0, 10, 20, 40, 80, 160 µM) treated HEB cells for 24 and 48 h. Scale bar = 50 µm. Data were presented as mean ± SD or as a representative image of triplicate experiments, *P<0.05, **P<0.01, ***P<0.001, ****P<0.0001 by one-way ANOVA with Tukey's post-hoc test.

The chemical structure of daphnoretin, identified by the CAS number 85622-93-1, with a molecular formula of C_19_H_12_O_7_ and a molecular weight of 352.3 Da, was depicted in Fig. 1A. The proliferation of HEB, U87 and U251 cells was assessed using the MTT assay across different concentrations (0, 10, 20, 40, 80, 100 μM) of daphnoretin, as depicted in Fig. 1B, C amd D. Microscopic examination after 24 h revealed a concentration-dependent inhibition of GBM cell proliferation by daphnoretin.

Furthermore, colony formation assays were conducted to evaluate the long-term inhibitory effects of daphnoretin on GBM cell proliferation. The results demonstrated a significant dose-dependent reduction in colony numbers in both U87 and U251 cells.

**Figure 2:**
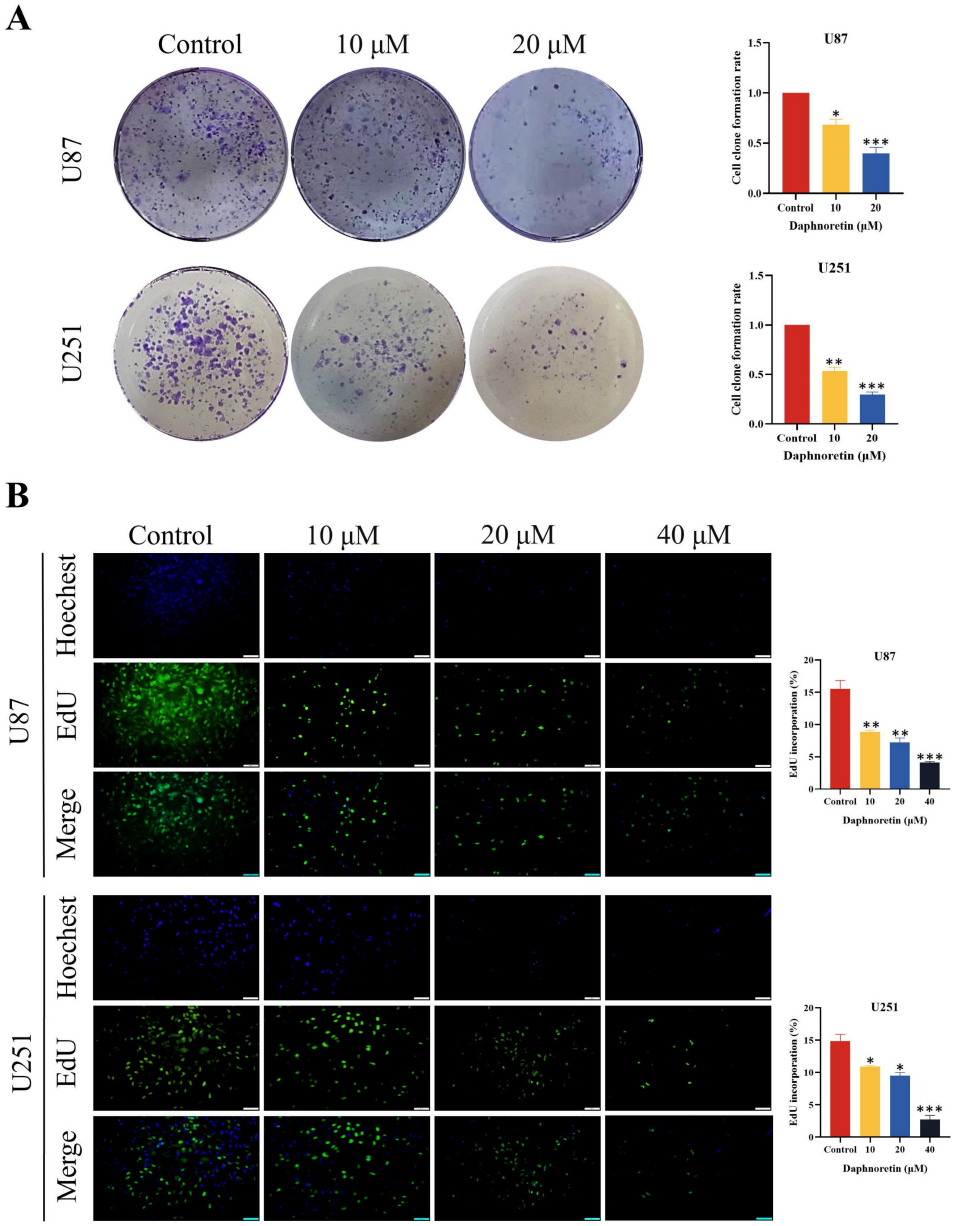
** Daphnoretin inhibits proliferation of GBM cells in dose-dependent manner.** (A) Clone formation assay using U87, U251 cells. (B) The cell proliferation of U87and U251 cells with indicated daphnoretin treatment were subjected to EdU assay. Scale bar = 100 µm. Data were presented as mean ± SD or as a representative image of triplicate experiments, *P<0.05, **P<0.01, ***P<0.001, ****P<0.0001 by one-way ANOVA with Tukey's post-hoc test.

To further validate the anti-proliferative capacity of daphnoretin, DNA synthesis was quantified using the EdU incorporation assay. Compared to the control group, daphnoretin-treated U87 and U251 cells exhibited a marked decrease in EdU-positive cells (Fig. 2B), indicating that daphnoretin effectively inhibits GBM cell proliferation.

Collectively, these findings underscore the potent anti-tumor effects of daphnoretin on GBM cells by impeding proliferation through multiple assays.

### 3.2 Daphnoretin inhibited GBM cells migration and invasion

The lateral migration ability of GBM cells was evaluated using a wound healing assay, as depicted in Fig. 2A. Following 24 hours of treatment with varying concentrations of daphnoretin, both U87 and U251 cells exhibited a significant dose-dependent decrease in migration ability. Compared to the control group, the gap distance widened progressively with increasing daphnoretin concentration, indicating effective inhibition of lateral migration by daphnoretin.

Additionally, migration and invasion capabilities of U87 and U251 cells were assessed using Transwell assays (Fig. 2B). After 24 hours of treatment, daphnoretin-treated cells showed marked reductions in both migration and invasion compared to untreated cells. These effects were dose-dependent, further demonstrating daphnoretin's ability to inhibit the migration and invasion of GBM cells.

### 3.3 Daphnoretin induces cell apoptosis in GBM cells

**Figure 4:**
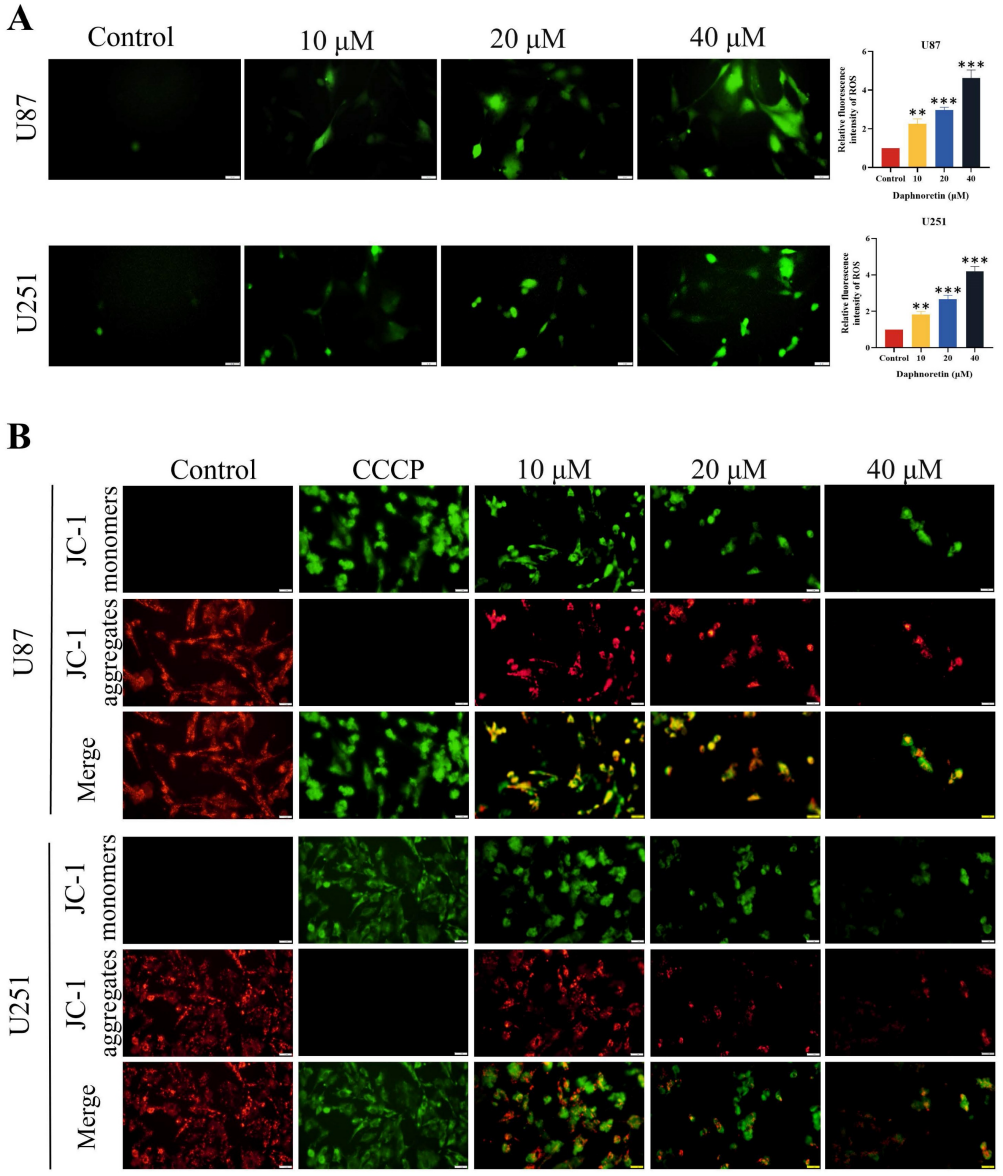
** Effects of daphnoretin on GBM cells ROS level and apoptosis.** (A) The ROS level was detected after U87 and U251 cells were treated with different concentrations of daphnoretin. Scale bar = 200 μm. (B) Fluorescence microscopy was used to detect changes in the mitochondrial membrane potential of U87 and U251 cells treated with daphnoretin for 24 h. Scale bar = 200 µm. Data were presented as mean ± SD or as a representative image of triplicate experiments, *P<0.05, **P<0.01, ***P<0.001, ****P<0.0001 by one-way ANOVA with Tukey's post-hoc test.

DCFH-DA staining was employed to assess the impact of daphnoretin on cellular ROS generation (Fig. 4A). Quantification of fluorescent images revealed a significant increase in ROS levels in cells treated with daphnoretin, indicating heightened oxidative stress.

The effect of daphnoretin on apoptosis in GBM cells was assessed using the mitochondrial membrane potential detection kit (JC-1), with results depicted in Fig. 4B. Carbonyl cyanide 3-chlorophenylhydrazone (CCCP), an apoptosis inducer that uncouples mitochondrial oxidative phosphorylation, served as a positive control to demonstrate mitochondrial membrane potential reduction. Increasing concentrations of daphnoretin resulted in an increase in the JC-1 monomer (green fluorescence) and a decrease in the JC-1 aggregate (red fluorescence), indicating an early apoptotic phenotype. These findings confirm that daphnoretin induces apoptosis in GBM cells.

**Figure 5:**
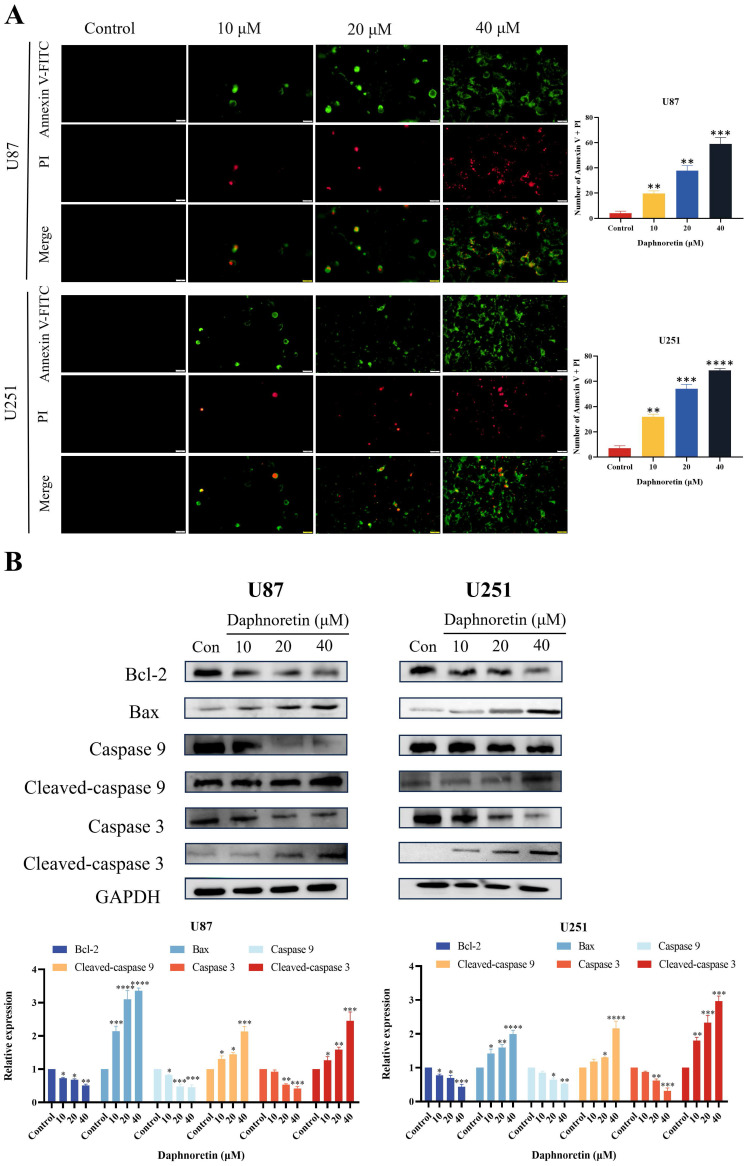
** Daphnoretin triggered mitochondria-dependent apoptosis in GBM cells.** (A) U87 and U251 cells were treated with the specified dose of daphnoretin for 24 h, stained with Annexin V-FITC/PI, and detected by fluorescence microscopy. Scale bar = 200 µm. (B) Western blot of Bcl-2, Bax, Caspase 9, Cleaved-caspase 9, Caspase 3 and Cleaved-caspase 3 in U87 and U251 cells treated with daphnoretin. Data were presented as mean ± SD or as a representative image of triplicate experiments, *P<0.05, **P<0.01, ***P<0.001, ****P<0.0001 by one-way ANOVA with Tukey's post-hoc test.

To elucidate the specific cell death mechanism induced by daphnoretin in GBM, Annexin V-FITC/PI staining was performed and observed under fluorescence microscopy (Fig. 5A). The proportion of apoptotic cells increased significantly with rising concentrations of daphnoretin.

Furthermore, the effect of daphnoretin on the expression levels of apoptosis-related proteins in U87 and U251 cells was examined using western blotting (Fig. 5B). After 24 hours of treatment, daphnoretin dose-dependently down-regulated Bcl-2 expression while up-regulating Bax expression. Moreover, there was a notable increase in the levels of Cleaved-caspase 9 and Cleaved-caspase 3, further supporting daphnoretin's induction of apoptosis in GBM cells.

### 3.4 Daphnoretin exerted anti-GBM activity *in vivo*

**Figure 6:**
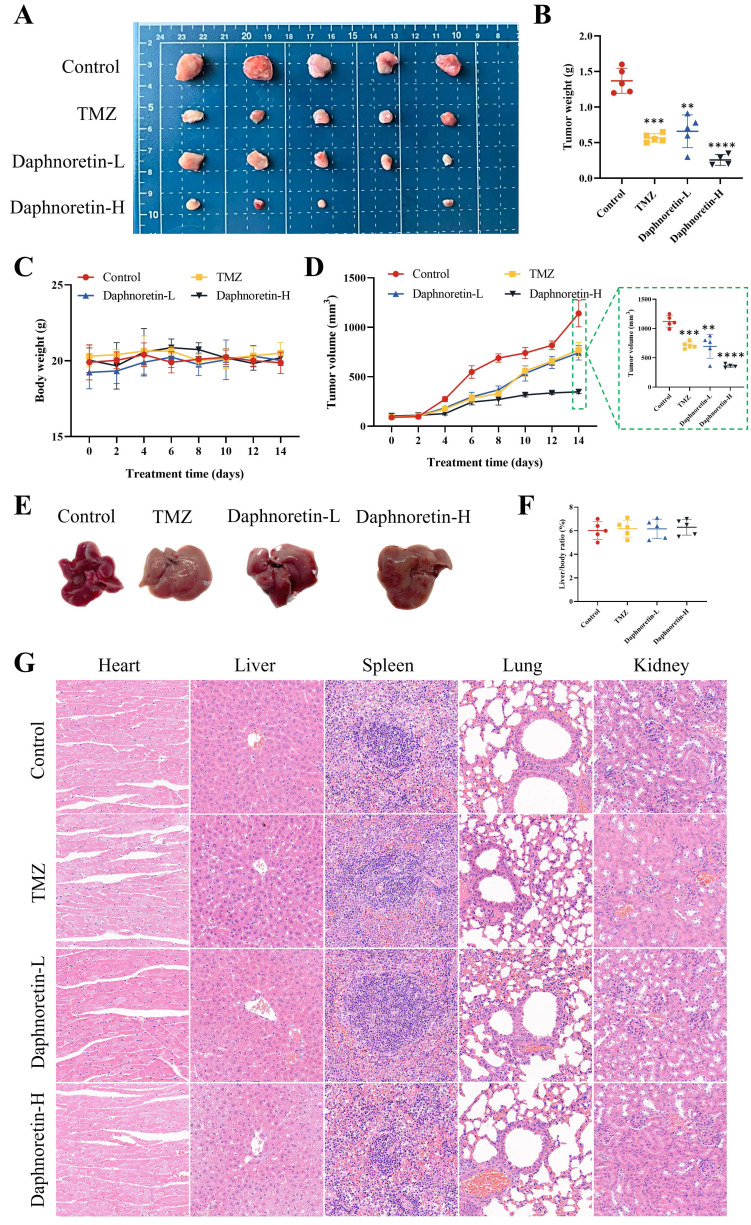
** Daphnoretin suppresses the tumor growth of GBM subcutaneous xenograft *in vivo*.** (A) Images of subcutaneous xenograft tumors in 4 groups (Control group, TMZ group, Daphnoretin-L group and Daphnoretin-H group). (B) Statistical graph of tumor weights in mice during treatment days. (C) Statistical graph of body weight in mice during treatment days. (D) Statistical graph of tumor volumes in mice during treatment days. (E) Liver images of the TMZ-treated and vary concentration of daphnoretin-treated xenograft mice. (F) Liver/body ratio of the TMZ-treated and vary concentration of daphnoretin-treated xenograft mice. (H) HE staining of heart, liver, spleen, lung and kidney of the TMZ-treated and vary concentration of daphnoretin-treated xenograft mice. Data were presented as mean ± SD or as a representative image, *P<0.05, **P<0.01, ***P<0.001, ****P<0.0001 vs. the control group by oneway ANOVA with Tukey's post-hoc test or two-way RM ANOVA with Sidak's multiple comparisons test.

To further confirm the inhibitory effect of daphnoretin on GBM in vivo, we administered daphnoretin in mouse xenograft models, with TMZ used as a positive control group. Results demonstrated that both low (Daphnoretin-L, 20 mg/kg) and high (Daphnoretin-H, 40 mg/kg) doses of daphnoretin significantly reduced tumor size, volume, and weight compared to the control group, as shown in Fig. 6A, B, and C, highlighting daphnoretin's promising anti-GBM efficacy in vivo. Assessing drug toxicity is crucial for effective drug development. Daphnoretin-treated mice exhibited no significant differences in liver morphology or liver/body ratio compared to the control group, as depicted in Fig. 6E and F. Furthermore, histological analysis of vital organs including the heart, liver, spleen, lung, and kidney revealed no notable abnormalities upon daphnoretin treatment (Fig. 6G, HE staining), indicating favorable biosafety of daphnoretin in vivo.

### 3.5 Daphnoretin might be a potential regulator of PI3K/AKT

**Figure 7:**
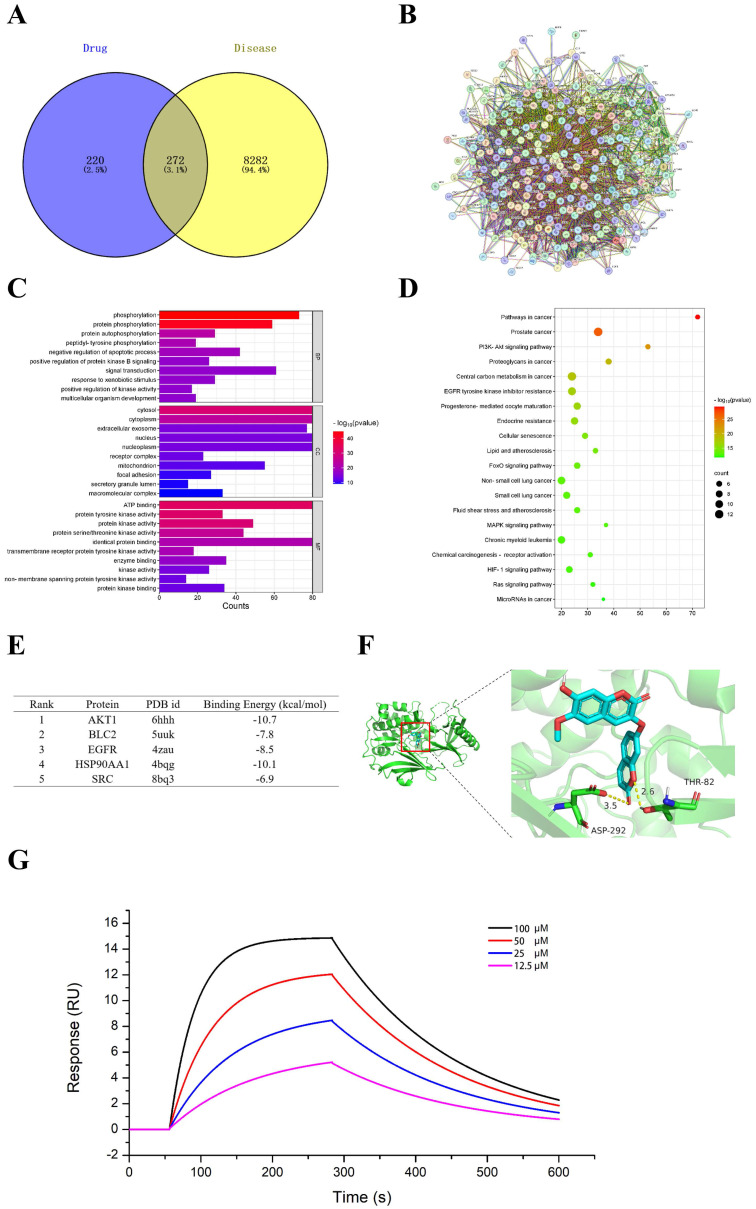
** Effects of daphnoretin on AKT based on network pharmacology and molecular docking analysis.** (A) The overlapped daphnoretin-GBM targets were screened out by Venny 2.1.0. (B) The PPI network of the Daphnoretin-GBM targets were conducted by STRING database. (C) The GO functional enrichments of overlapped Daphnoretin-GBM targets were analyzed by R (v.3.6.3) software. (D) The KEGG pathway enrichments of overlapped Daphnoretin-GBM targets were performed by R (v.3.6.3) software. (E) The binding energy with PDB id of 5 combining conformations. The smaller of the binding energy value, the higher the accuracy. (F) Molecular docking results of daphnoretin with AKT1 was obtained by Autodock-VINA and Pymol software. (G) Different concentrations of daphnoretin associated with AKT as measured by SPR analysis. Data were presented as mean ± SD or as a representative image, *P<0.05, **P<0.01, ***P<0.001, ****P<0.0001 vs. the control group by oneway ANOVA with Tukey's post-hoc test or two-way RM ANOVA with Sidak's multiple comparisons test.

The mechanism of daphnoretin's inhibition of GBM cell proliferation was initially explored using network pharmacology. Four hundred ninety-two potential targets of daphnoretin were identified through GeneCards and SwissTargetPrediction databases, while 8,554 targets associated with GBM were predicted from comprehensive analyses of GeneCards and TCGA databases. A Venn diagram depicting the overlap between daphnoretin and GBM targets revealed 272 shared targets, suggesting potential targets for daphnoretin treatment against GBM (Fig. 7A). Protein-protein interaction (PPI) analysis identified AKT1, VEGFR, HSP90AA1, BCL2, and SRC as hub genes potentially pivotal in the treatment of GBM (Fig. 7B). GO enrichment analysis of biological processes (BP), cellular components (CC), and molecular functions (MF) among common drug-disease targets, along with top ten disease targets, conducted via Cytoscape 3.8.0, indicated that intracellular redox states, such as phosphorylation and protein phosphorylation, played critical roles in GBM under daphnoretin treatment (Fig. 7C). KEGG pathway analysis highlighted the PI3K-AKT signaling pathway as central to daphnoretin's effects against GBM (Fig. 7D).

Additionally, molecular docking of five key target structures with daphnoretin revealed binding energies (kcal/mol) that demonstrated stable ligand-receptor interactions, with lower values indicating stronger binding (Fig. 7E). To validate the above hypothesis, the interaction between daphnoretin and AKT was first evaluated using SPR analysis, and daphnoretin was injected over immobilized recombinant human AKT on a chip surface to determine the kinetics of the binding reaction. Visualization of molecular docking analysis between daphnoretin and the PI3K/AKT pathway (Fig. 7G) underscored a robust binding affinity, suggesting daphnoretin's potential to target PI3K/AKT-related pathways involved in cell apoptosis and GBM attenuation, as indicated by network pharmacology and docking analyses.

### 3.6 Effect of Daphnoretin on the PI3K/AKT pathway

**Figure 8:**
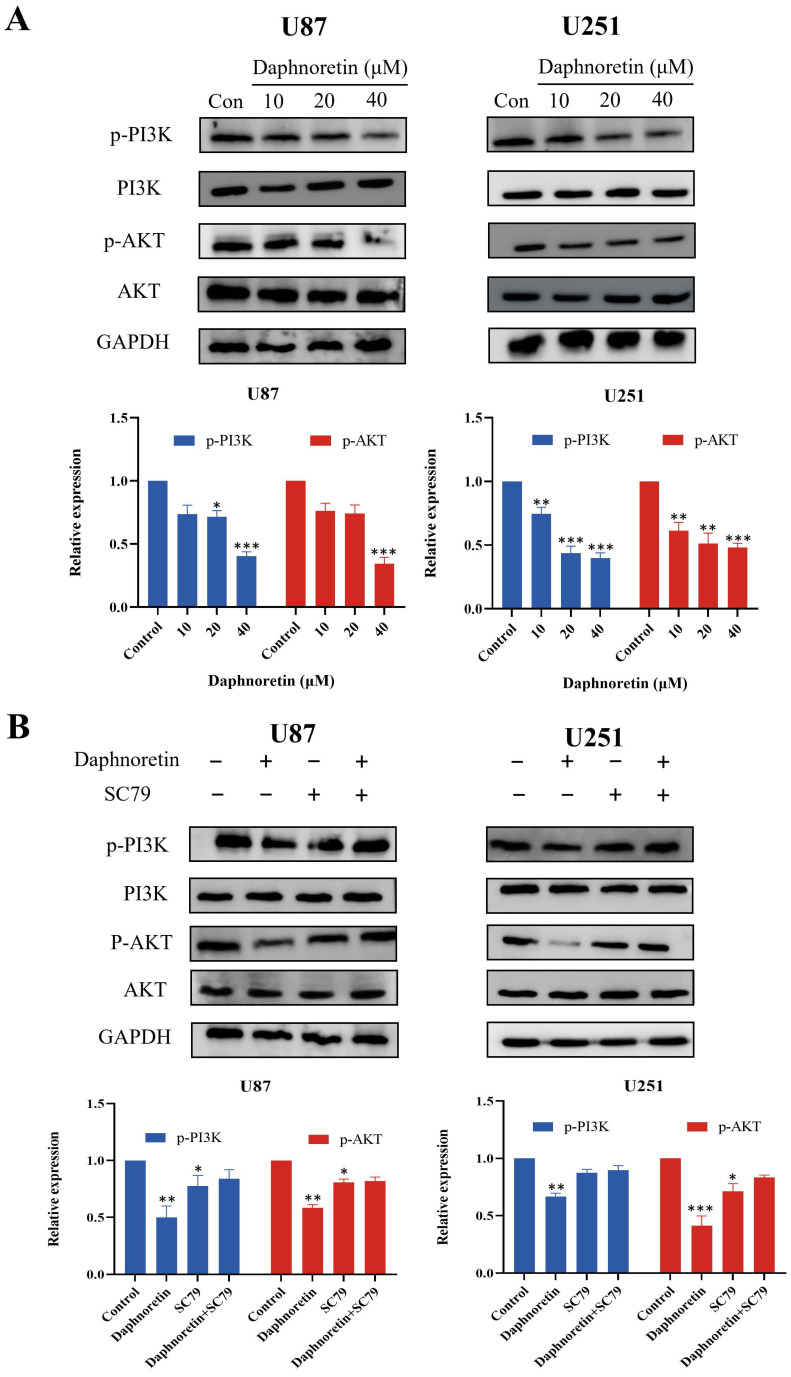
** Daphnoretin triggers PI3K/AKT-dependent apoptotic pathway in vitro.** (A) U87 and U251 cells were treated with increasing doses of daphnoretin, and the proteins related to PI3K / AKT signaling pathway were analyzed by Western blot. (B) After pretreatment with SC79 for 1 h, the expression levels of PI3K, p-PI3K, AKT and p-AKT. Data were presented as mean ± SD or as a representative image, *P<0.05, **P<0.01, ***P<0.001, ****P<0.0001 vs. the control group by oneway ANOVA with Tukey's post-hoc test or two-way RM ANOVA with Sidak's multiple comparisons test.

Based on the pivotal regulatory role of PI3K/AKT signaling molecules in cell proliferation, we assessed the levels of these molecules in daphnoretin-treated GBM cells. Our findings revealed significant downregulation of p-PI3K and p-AKT levels in U87 cells following daphnoretin treatment, suggesting daphnoretin's ability to modulate U87 cell proliferation via the PI3K/AKT pathway. Similarly, decreased levels of p-PI3K and p-AKT were observed in daphnoretin-treated U251 cells (Fig. 8A).

To further investigate the role of the PI3K/AKT pathway, we conducted rescue experiments using SC79, an AKT agonist. Pretreatment with SC79 for 1 hour abolished the effects of daphnoretin (20 μM) on cell proliferation, apoptosis, and PI3K/AKT signaling in GBM cells (Fig. 8B). These results underscore the essential role of the PI3K/AKT signaling cascade in mediating daphnoretin's anti-tumor effects in GBM cells.

**Figure 9:**
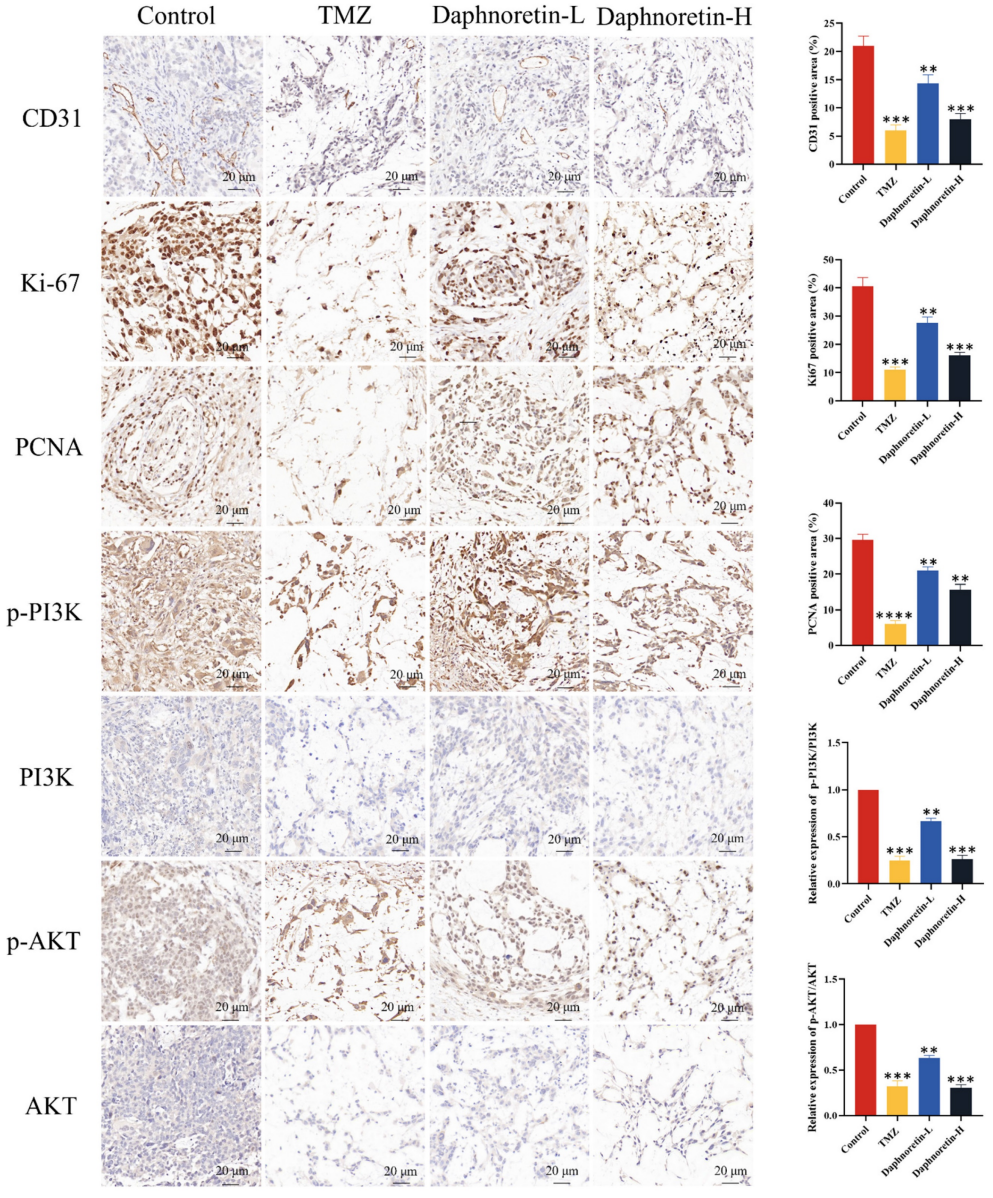
** Daphnoretin suppresses the PI3K/AKT signaling pathway *in vivo*.** (A) IHC staining with antibody against CD31, Ki-67, PCNA, p-PI3K, PI3K, p-AKT and AKT of tumor tissues. (B) The statistical results of positive areas. And the expressions of phosphorylated proteins are normalized to comparison with their non-phosphorylated counterparts. Scale bar = 50 μm. The data were expressed as mean ± SD. The experiment was repeated three times. After one-way analysis of variance and Tukey's post-hoc test, compared with the control group: *P<0.05, **P<0.01, ***P<0.001, ****P<0.0001.

Additionally, IHC analysis was performed to assess cell proliferation (Ki-67 and PCNA), apoptosis (p-PI3K, PI3K, p-AKT, AKT), and tumor angiogenesis (CD31). Figure 9 demonstrates a significant decrease in Ki-67, PCNA, and CD31 expression levels, accompanied by an increase in p-PI3K and p-AKT expression levels with increasing doses of daphnoretin. These findings indicate that daphnoretin promotes apoptosis, inhibits cell proliferation, and suppresses tumor angiogenesis in vivo.

## 4. Discussion

GBM stands out as one of the most aggressive and lethal malignant tumors affecting the central nervous system. The treatment of GBM has long posed a significant challenge in neuro-oncology 10, 11. Despite advancements in comprehensive treatment modalities such as surgery, radiotherapy, and chemotherapy in recent years, the prognosis for GBM patients remains poor, with an extremely low 5-year survival rate 12. Therefore, identifying new therapeutic targets and drugs to enhance the efficacy of GBM treatment is of paramount importance.

Daphnoretin has demonstrated antitumor activity against various cancer cell lines including colon cancer HCT116 cells, cervical cancer HeLa cells, oral epidermoid carcinoma KB cells, lung cancer A549 cells, and NCI-H187 cells 13-16. In our study, we observed that daphnoretin significantly inhibits proliferation, migration, and invasion while inducing apoptosis in GBM cells, effects that are closely associated with the PI3K/AKT pathway.

Apoptosis represents a critical and multistep biological process essential for maintaining cellular homeostasis 17, 18. Key regulators of apoptosis include the pro-apoptotic Bax family proteins and the antiapoptotic Bcl-2 family molecules. The balance between Bcl-2 and Bax levels serves as a crucial indicator of cancer cell apoptosis induced by chemotherapy and radiation 18. Disruption of this balance alters mitochondrial membrane permeability (MMP), leading to cytochrome C release into the cytoplasm and subsequent upregulation of Cleaved-caspase 9 and Cleaved-caspase 3 levels 20-22. In our study, daphnoretin significantly increased apoptotic rates in GBM cells. We observed downregulation of Bcl-2 levels and upregulation of Bax, Cleaved-caspase 9, and Cleaved-caspase 3 levels in both daphnoretin-treated U87 and U251 cells. These findings suggest that daphnoretin induces cell apoptosis through the mitochondrial apoptotic pathway in GBM. Daphnoretin triggers programmed cell death in cancer cells through the activation of the intrinsic apoptotic pathway. It upregulates pro-apoptotic factors such as Bax and downregulates anti-apoptotic proteins like Bcl-2, leading to mitochondrial dysfunction and activation of caspases. It impairs cell cycle progression by influencing cyclins and cyclin-dependent kinases, thereby halting cancer cell proliferation at various phases of the cell cycle 5, 6.

Daphnoretin is known to exert potent anti-tumor effects through various signaling cascades, including MEK, PI3K/AKT, and NF-κB pathways 23. In the field of anti-cancer, daphnoretin showed strong anti-tumor activity. Studies have shown that daphnetin can significantly inhibit the proliferation of a variety of tumor cells. Its anti-cancer mechanism mainly involves the regulation of key signaling pathways. For example, daphnoretin can play a tumor suppressor role by up-regulating the expression of p53 protein. p53 is an important tumor suppressor gene, which inhibits tumor growth by regulating cell cycle, promoting tumor cell senescence and apoptosis, inhibiting angiogenesis and tumor metastasis 24. Further studies revealed that daphnetin may inhibit the differentiation of osteoclasts through the NF-κB pathway, thereby exerting anti-inflammatory effects. NF-κB is an important transcription factor, which is involved in the regulation of the expression of a variety of inflammation-related genes 25. Daphnetin inhibits the activity of NF-κB by promoting the expression of IκB-α (NF-κB), thereby down-regulating the expression of transcription factors NFATc-1 and c-Fos closely related to osteoclast differentiation, and ultimately inhibiting osteoclast differentiation.

The PI3K/AKT signaling cascade plays a pivotal role in regulating cell growth, differentiation, and is frequently dysregulated in various human cancers, including GBM 27, 28. Activation of the PI3K/AKT pathway promotes GBM development by inhibiting apoptosis, accelerating the cell cycle, enhancing tumor cell proliferation, and facilitating metastasis 27, 28. Moreover, the PI3K/AKT pathway serves as a target for numerous anti-tumor drugs and is closely associated with treatment response and resistance in various cancers 29, 30. SC79 is a small molecule that acts as an allosteric activator of AKT. By binding to AKT, SC79 promotes its phosphorylation at Thr308 and Ser473, thereby enhancing Akt's enzymatic activity and promoting its anti-apoptotic effects. SC79 binds to Akt, facilitating its phosphorylation by upstream kinases such as PI3K. This leads to full activation of Akt, which subsequently phosphorylates downstream targets involved in cell survival and proliferation 31-33.

In the current study, Western blot data demonstrated that daphnoretin downregulates p-PI3K and p-AKT levels in U87 and U251 cells. Rescue experiments using the AKT activator SC79 significantly reversed daphnoretin's anti-tumor effects on GBM cells, inhibiting proliferation and metastasis, inducing apoptosis, and downregulating the PI3K/AKT signaling pathway in vitro. These results collectively indicate that daphnoretin modulates apoptosis and metastasis in GBM by targeting the PI3K/AKT signaling pathway. To assess its efficacy in vivo, we established a xenograft model to investigate daphnoretin's anti-tumor effects. We confirmed that daphnoretin inhibits tumor growth in a dose-dependent manner without causing substantial weight loss in the experimental groups, suggesting its safety and absence of significant side effects in vivo. Immunohistochemical (IHC) assays further supported that daphnoretin exerts inhibitory effects through mechanisms consistent with those observed in vitro, specifically via downregulation of the PI3K/AKT signaling cascades in GBM tumors. In addition, our study has limitations to consider. It primarily focused on daphnoretin treatment in mouse models rather than human tissues, which is a notable constraint. Future research should aim to elucidate the specific targeting relationship between daphnoretin and PI3K/AKT, exploring potential targeting sites in human GBM tissues. Overall, daphnoretin shows promise as a novel small molecule therapeutic agent for treating GBM.

## 5. Conclusion

In summary, our findings reveal that daphnoretin effectively inhibits proliferation, migration, and invasion of GBM cells in vitro in a dose-dependent manner. In vivo studies demonstrate that daphnoretin suppresses tumor growth without causing significant drug toxicity. Mechanistic investigations underscore that daphnoretin induces apoptosis in glioblastoma cells via the PI3K/AKT signaling pathway. The intricate details of daphnoretin's anti-cancer mechanism warrant further exploration in future studies.

## Figures and Tables

**Figure 3:**
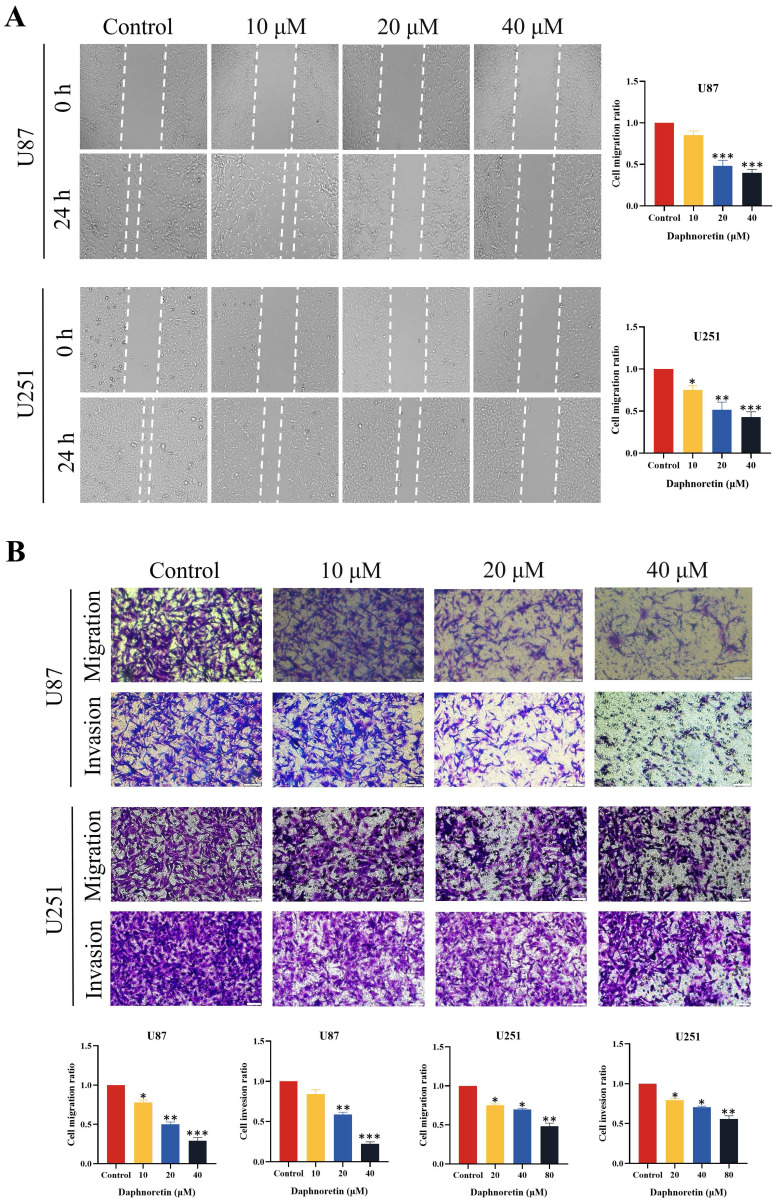
** Daphnoretin inhibits migration and invasion of GBM cells in dose-dependent manner.** (A) Measurement of wound healing in U87 and U251 cells exposed to daphnoretin. Scale bar = 50 µm. (B) The vertical migration and invasion of U87 and U251 cells after daphnoretin treatment was measured by Transwell assay. Scale bar =200 µm. Data were presented as mean ± SD or as a representative image of triplicate experiments, *P<0.05, **P<0.01, ***P<0.001, ****P<0.0001 by one-way ANOVA with Tukey's post-hoc test.

**Figure 10:**
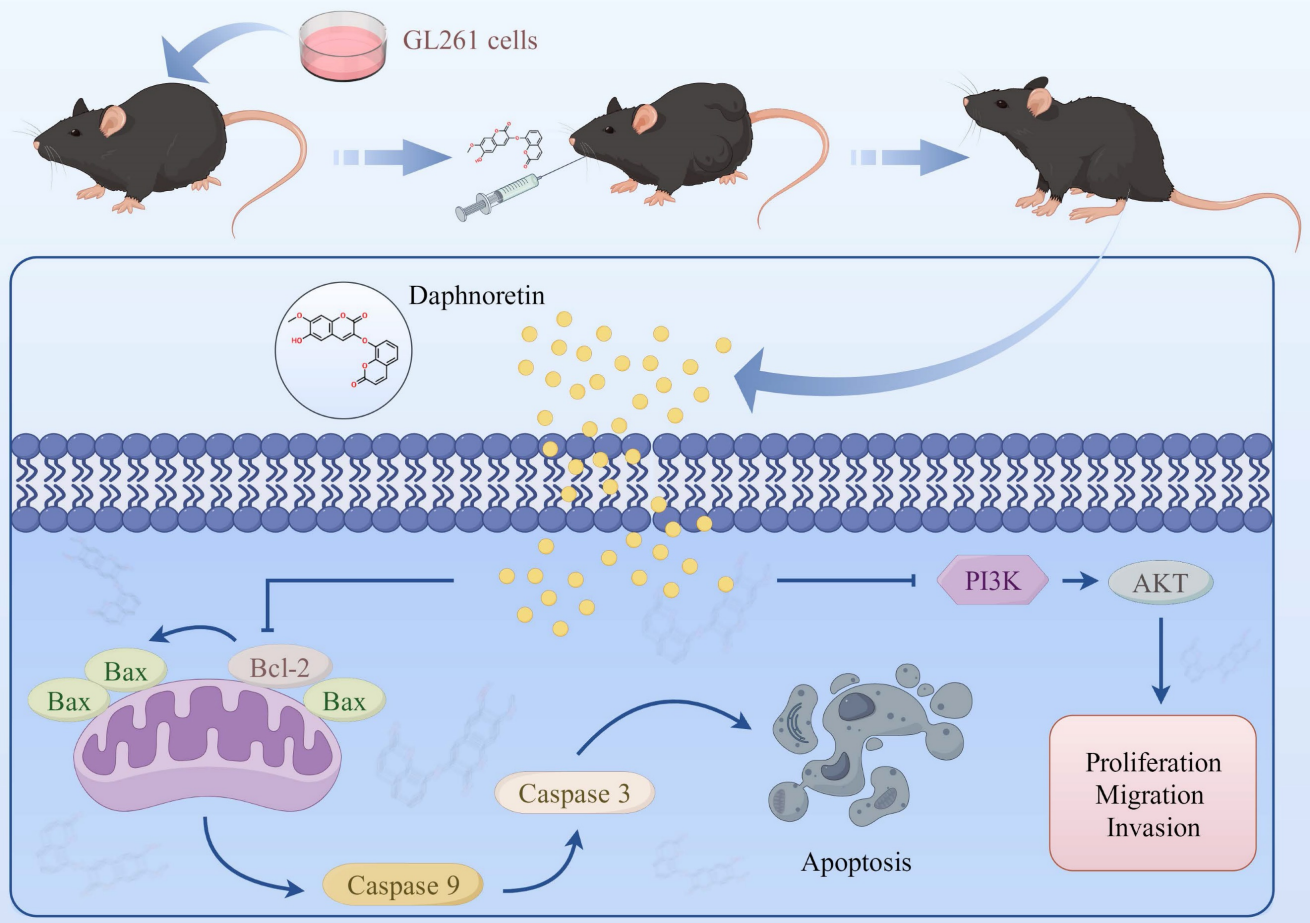
Schematic model of the proposed mechanism shows how daphnoretin induces apoptosis in GBM and the possible via PI3K/AKT signaling pathway.
